# Stereotype Threat and Perceptions of Family-Friendly Policies among Female Employees

**DOI:** 10.3389/fpsyg.2016.02043

**Published:** 2017-01-05

**Authors:** Courtney von Hippel, Elise K. Kalokerinos, Hannes Zacher

**Affiliations:** ^1^School of Psychology, University of Queensland, BrisbaneQLD, Australia; ^2^Faculty of Psychology and Educational Sciences, KU LeuvenLeuven, Belgium; ^3^Institute of Psychology, University of LeipzigLeipzig, Germany

**Keywords:** family-friendly policies, gender, stereotype threat, work-life balance, work-family interface

## Abstract

In their efforts to recruit and retain female employees, organizations often attempt to make their workplaces “family-friendly.” Yet there is little research on how women view family-friendly policies, particularly women who experience gender-based stereotype threat, or the concern of being viewed through the lens of gender stereotypes at work. Pilot research with female managers (*N* = 169) showed that women who experienced stereotype threat perceived more negative career consequences for utilizing family-friendly policies. We then conducted two studies to further probe this relationship. Study 1 replicated the relationship between stereotype threat and the perceived consequences of utilizing family-friendly policies among women who recently returned to work after the birth of a child (*N* = 65). In Study 2 (*N* = 473), female employees who reported feelings of stereotype threat perceived more negative consequences of utilizing family-friendly policies, but they also reported greater intentions to use these policies. Our findings suggest that female employees are susceptible to stereotype threat, which in turn is associated with more negative views of family-friendly policies. Thus, the mere provision of such policies may not create the kind of family-friendly workplaces that organizations are attempting to provide.

## Introduction

Concerns about gender equity, recruitment, and retention push firms to provide flexible scheduling and alternative work arrangements, broadly known as work-life balance practices or family-friendly policies (Beauregard and Henry, 2009). Family-friendly practices include a large range of programs that often focus on issues of flexible scheduling (e.g., flextime, compressed work week, telework) and support for family care (e.g., parental leave, compassionate leave, on-site childcare). Any policy or program designed to help employees balance work and family (or non-work) constitutes a family-friendly policy. Thus, stress management programs, eldercare support, and providing a lactation room for mothers are all examples of family-friendly policies. Family-friendly policies give organizations an advantage when they compete for employees, with these policies shown to positively impact recruitment and retention (see Beauregard and Henry, 2009 for a review of family-friendly policies and their impact on organizational performance). They are also aligned with pressure on firms to enact policies that help women work while raising a family, which have been prioritized by governments as fertility rates have fallen below replacement levels in most industrialized nations (Lesthaeghe, 2014).

At first blush, family-friendly policies appear to be an ideal solution to gender-equity concerns in the workplace. For example, these policies can enable women to continue in caregiving roles without being forced to side-track or derail their career. Nevertheless, unexpected trade-offs between well-intended human resource management practices and important employee outcomes, such as occupational well-being are not uncommon (Grant et al., 2007; Kaiser et al., 2013). Family-friendly policies may be one such practice, providing opportunities for women to better balance work and family, but at a potential cost if those who utilize these policies worry there will be negative career consequences of doing so. In this article, we report pilot data and two studies designed to investigate how experiences of stereotype threat relate to female workers’ perceptions of the career consequences of utilizing family-friendly policies.

## Stereotype Threat and Family-Friendly Policies

Women working in the corporate world often experience stereotype threat (e.g., von Hippel et al., 2015; Hoyt and Murphy, 2016), which is the concern that they are being evaluated through the lens of negative gender stereotypes (Steele, 1997). Women in corporate environments are often in positions in which their job requires characteristics that are inconsistent with their gender identity (Heilman and Okimoto, 2007). For example, a senior manager should be analytical, independent, and assertive, as these (stereotypically masculine) traits are associated with managerial success. In contrast, stereotypic female traits – being gentle, warm, and tender – are seen as inconsistent with the traits required for success in most businesses (e.g., Koenig et al., 2011). Thus, women are often required to manage their contrasting “female” and “work” identities in order to emphasize their role as skilled employees, particularly when such skills are counter-stereotypic for women. Actor, writer, and producer Rashida Jones encapsulates this struggle to manage her multiple identities: “I want to be a boss and also be vulnerable. I want to be outspoken and respected, but also sexy and beautiful” (New York Times, 2016 October 18). Efforts to manage these competing identities can result in stereotype threat, as women become acutely sensitive to the possibility that they are being stereotyped (von Hippel et al., 2011a). Importantly, it is not necessary to actually be stereotyped by others to experience stereotype threat, nor must people believe the stereotype is true of their group or themselves. Rather, people need only worry that they may be stereotyped for stereotype threat effects to emerge (Steele, 1997).

In Steele’s (1997) original theoretical description of stereotype threat, he discussed two types of consequences: acute performance deficits and attitudinal consequences. A large body of research has now demonstrated the performance impairing effects of stereotype threat in the laboratory (for a meta-analysis, see Nguyen and Ryan, 2008), but far less research has examined the attitudinal consequences of stereotype threat (Kalokerinos et al., 2014; Kulik et al., 2016). For example, when people experience stereotype threat they report lowered aspirations, view the stereotyped domain as less important to their self-concept, indicate less interest in participating in the stereotyped domain, and attempt to disassociate themselves from the stereotyped domain (Steele and Aronson, 1995; Davies et al., 2005; Murphy et al., 2007). Although research examining the effects of stereotype threat on *organizational outcomes* such as job attitudes and disengagement is still in its infancy, the studies that do exist are consistent with Steele’s argument that stereotype threat can have long-term consequences on domain-relevant attitudes (e.g., von Hippel et al., 2013).

The finding that women are susceptible to stereotype threat in the workplace is not surprising, given the incongruity described above between the female gender role (e.g., being nurturing, kind, sensitive) and the masculine traits required for success in many organizations (e.g., being achievement-oriented, competitive, dominant; Eagly and Karau, 2002; Schein, 2007). Indeed, experimental work in organizational settings has demonstrated that stereotype threat causes female employees to respond to these competing role demands by separating their work and gender identities, apparently as a way to manage internal conflict between these competing identities (von Hippel et al., 2011b).

In addition to such intra-personal responses to the role conflict induced by stereotype threat, women may also engage in various inter-personal strategies to manage stereotype threat and minimize the associated negative perceptions of others. For example, members of stereotyped groups have been shown to claim disinterest in activities that are stereotypic of their group (Steele and Aronson, 1995) and to claim competence in counter-stereotypic domains (von Hippel et al., 2005). People in such circumstances also assert that stereotypic qualities of their group are not self-descriptive (Pronin et al., 2004). It seems possible that these processes of distancing oneself from the stereotype may play a role in women’s responses to the opportunities provided by family-friendly policies.

By virtue of the fact that family-friendly policies are generally directed at and adopted by women (Sabattini and Crosby, 2009), they have the potential to cast their recipients as stereotypically female and in need of help. When women, particularly those who experience stereotype threat, are already trying to manage the impressions held by their colleagues, they may view such implications of family-friendly policies as undermining their efforts to distance themselves from the stereotype of their group. Because women are stereotyped as less committed to their careers and more focused on their families (Correll et al., 2007), they may worry that if they avail themselves of family-friendly policies they will only be confirming the very stereotype they are endeavoring to refute. Thus, to the degree that women experience stereotype threat, they may believe there are negative career consequences from using family-friendly policies, and may be reticent to utilize the very policies that have been designed to help them. Moreover, because susceptibility to stereotype threat is an indication that women feel they are undervalued in their firm by virtue of their female-stereotypic qualities, these women may be concerned that utilizing family-friendly policies may be seen as confirming these gender stereotypes, and thus exacerbate this problem. Thus, we hypothesize that the women who experience stereotype threat will be more likely to perceive negative career consequences of using family-friendly policies.

In this paper, we present studies using experimental (pilot study) and cross-sectional designs (Studies 1 and 2) to explore this possibility. We predict that stereotype threat will be associated with perceptions that using family-friendly policies has negative career consequences. As a consequence, we also hypothesize that stereotype threat will be negatively associated with interest in using family-friendly policies.

This research contributes to the literatures on the work-family interface and stereotype threat by highlighting the potential unintended negative consequences of family-friendly policies (i.e., perceiving policy utilization as potentially harmful to one’s career). The work-family literature has not yet examined the role of stereotype threat in understanding the effects of organizational interventions on employee outcomes. Moreover, while research on stereotype threat in organizations is growing, few studies have examined outcomes other than job performance (Kalokerinos et al., 2014; Walton et al., 2015).

## Pilot Study

In von Hippel et al. (2011b), we manipulated an antecedent of stereotype threat to investigate the causal role of stereotype threat in identity separation among female managers. The proportion of women in a performance context has been shown to affect feelings of stereotype threat and concomitant performance (Sekaquaptewa and Thompson, 2002; Inzlicht and Ben-Zeev, 2000; Murphy et al., 2007). Thus, in that study, we manipulated the accessibility of male-dominance in the workplace by either reminding or not reminding female managers that most partners in their firm are male. By increasing the accessibility of the gender imbalance in their organization, this reminder was intended to induce stereotype threat.

The corporation in which we conducted this study was interested in their employees’ perceptions of their family-friendly programs, which focused on flexible scheduling (e.g., flexible work options, such as flextime) and alternative career paths (e.g., job sharing, telework). Thus, in collaboration with the organization we added items to the survey to assess the perceived career consequences of utilizing family-friendly policies, as well as perceived organizational support for family-friendly policies. Although these items were originally developed in collaboration with the organization, they are nevertheless of potential interest in the context of stereotype threat. When women experience stereotype threat at work they may worry that utilizing family-friendly policies will reinforce or confirm the very stereotypes they are endeavoring to refute. As a consequence, women who experience stereotype threat may be particularly likely to believe that utilizing family-friendly policies is harmful to their careers (*Hypothesis 1*). We further predict that a reminder about the male dominance in the upper echelons of the firm leads to feelings of stereotype threat, which in turn causes women to believe utilizing family-friendly work arrangements are harmful to their careers (*Hypothesis 2*). Finally, we explore the possibility that perceived organizational support mediates the relationship between the gender imbalance reminder and beliefs that utilizing family-friendly policies has negative career consequences.

### Method

#### Participants and Design

As noted above, these pilot data were part of a larger data collection effort, and the remaining data have been previously published (von Hippel et al., 2011b). Participants were female senior managers working in the Australian office of an international accounting and consulting firm. Initially, 188 participants opened the survey, and of these participants, 169 completed the measures of stereotype threat and evaluations of family-friendly policies. Around half of the participants (42.3%) reported having children. To help ensure anonymity of participants, age was assessed categorically: 13.2% of participants were 25 to 29 years of age, 35.9% of participants were 30 to 34, 26.9% were 35 to 39, 18.0% were 40 to 44, 4.2% were 45 to 49, and 1.8% were 50 or older. The average tenure for women in the organization was 6.9 years (*SD* = 4.7).

Participants were randomly assigned to one of two experimental conditions when they accessed the online survey. The stereotype threat manipulation was adapted from Murphy et al. (2007), with approximately half of the participants (*N* = 81) reminded of the percentage of females in the firm (coded as 1). These participations read an introduction saying “*Thank you for choosing to complete this questionnaire seeking to understand why only 10% of the partners at [firm name] are females*. The participants (*N* = 88) in the control condition (coded as -1) read an introduction saying “*Thank you for choosing to complete this questionnaire investigating employees’ self-concepts, goals, and experiences at work*.”

#### Measures

Responses were provided on 7-point scales, ranging from 1 = *strongly disagree* to 7 = *strongly agree*. Because the organization had a well-known family-friendly policy program in place, we referred to this specific program and did not need to define the construct for participants.

##### Stereotype threat

To check that the manipulation successfully elicited feelings of stereotype threat, we used a single item from von Hippel et al. (2011a): “*Some of my colleagues feel that I have less managerial ability because I’m a woman.*” This item is intended to tap participants’ concern that others may discount their ability due to their gender, and was itself adapted from Steele and Aronson℉s (1995) three-item scale measuring stereotype threat. After answering this question, participants completed measures unrelated to the current manuscript that were previously reported in von Hippel et al. (2011b).

##### Perceived negative career consequences of utilizing family-friendly policies

Perceived negative career consequences of utilizing family-friendly policies were assessed with the single item “*Women who accept family-friendly policies limit their career opportunities.*”

##### Perceived organizational support for family-friendly policies

Perceived organizational support for family-friendly policies was assessed with the single item “*Family-friendly policies are not supported in day-to-day practice*,” which was adapted from Eisenberger et al.’s (1990) scale on perceived organizational support. This item was reverse-coded such that higher numbers indicated greater perceived organizational support.

### Results and Discussion

**Table 1** provides the descriptive statistics and correlations. An independent groups *t*-test indicated that those in the gender imbalance condition scored significantly higher on the stereotype threat measure (*M* = 3.48, *SD* = 1.98) than those in the control condition (*M* = 2.70, *SD* = 1.72), *t*(167) = 2.74, *p* = 0.007, Cohen’s *d* = 0.42. Measured stereotype threat was positively associated with perceived negative career consequences of utilizing family-friendly policies, supporting Hypothesis 1 (see **Table 1**).

**Table 1:** Descriptive statistics and correlations for pilot study.

Variable	1	2	3	4	*M*	*SD*
1. Stereotype threat	-	0.21^∗∗^	-0.28^∗∗∗^	0.07^∗^	3.08	1.88
2. Perceived negative career consequences of utilizing FFP		-	-0.34^∗∗∗^	0.02	5.02	1.74
3. Perceived organizational support for FFP			-	0.09	3.63	1.99
4. Parental status					0.58	0.50


*FFP, family-friendly policies. Parental status is coded as -1 = no children, 1 = children. All correlations use pairwise deletion (*N* varies between 167 and 169). ^∗^*p* < 0.05; ^∗∗^*p* < 0.01; ^∗∗∗^*p* < 0.001.*

An independent groups *t*-test revealed no difference in the perceived negative career consequences of utilizing family-friendly policies between the gender imbalance (coded as 1; *M* = 5.22, *SD* = 1.59) and control (coded as -1; *M* = 4.84, *SD* = 1.85) conditions, *t*(166.22) = 1.44, *p* = 0.152, Cohen’s *d* = 0.22^1^.

An independent groups *t*-test revealed that participants in the gender imbalance condition perceived lower organizational support for family-friendly policies (*M* = 3.16, *SD* = 1.85) than those in the control condition (*M* = 4.06, *SD* = 2.04), *t*(166.97) = 3.00, *p* = 0.003, Cohen’s *d* = 0.46.^2^ This finding suggests a direct effect of gender imbalance on perceived organizational support for family-friendly policies. Across the whole sample (*M* = 3.63, *SD* = 1.99), stereotype threat was negatively associated with perceived organizational support for these policies (*r* = -0.28, *p* = 0.001).

**FIGURE 1:**
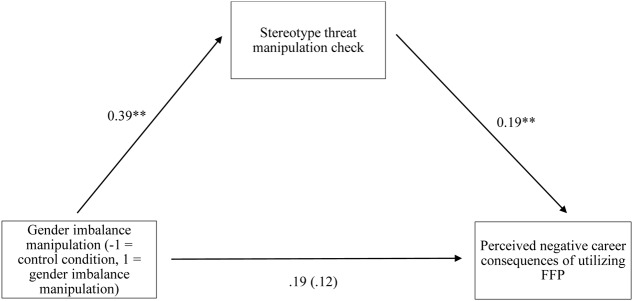
**Model of the relationships between the gender imbalance reminder manipulation, stereotype threat, and the perceived career consequences of utilizing family-friendly policies (FFP).** Numbers are unstandardized coefficients. Coefficients in brackets indicate weight after inclusion of mediators. ^∗^*p* < 0.05; ^∗∗^*p* < 0.01; ^∗∗∗^*p* < 0.001.

To examine the hypothesis that the gender imbalance reminder would indirectly affect the perceived career consequences of family-friendly policies via stereotype threat, we conducted bootstrapped mediation analyses with 10,000 resamples (using model 4 of the PROCESS macro; Hayes, 2008). This analysis revealed that the indirect effect of the gender imbalance reminder through stereotype threat was significant (see **Figure 1**; *IE* = 0.07, *SE* = 0.04, 95% CI: 0.02, 0.18)^3^. Thus, Hypothesis 2 was supported.

Exploratory analyses examining perceptions of organizational support for family-friendly policies were also conducted. Bootstrapped mediation analyses testing the indirect effect of the gender imbalance reminder on the perceived organizational support for family-friendly policies via stereotype threat revealed that the indirect effects of the gender imbalance reminder through stereotype threat was significant (*IE* = -0.09, *SE* = 0.05, 95% CI: -0.23, -0.02)^4^. Interestingly, there was a significant indirect effect of the gender imbalance manipulation on perceived consequences through perceived organizational support (*IE* = 0.13, *SE* = 0.06, 95% CI: 0.04, 0.27).

The results of this pilot study demonstrate that a reminder about male dominance in the upper echelons of the firm induced stereotype threat (as previously reported in von Hippel et al., 2011b), which in turn led women to believe utilizing family-friendly work arrangements would be harmful to their careers. Exploratory analyses suggest that one explanation for these findings may be that a small proportion of women in the upper echelons of the organization leads to perceptions that the organization does not really support family-friendly policies in day-to-day practice, which in turn is associated with perceived negative career consequences of policy utilization. Interestingly, although the policies offered by the organization are couched as “family-friendly,” as can be seen in the footnotes the results remained unchanged whether women had children living at home or not. This finding suggests that women may contend with the stereotype that they must manage work and family, irrespective of whether they have children. Such a possibility is unsurprising given that women do more housework than men (Bianchi et al., 2012) and are more likely to care for elderly parents (Van Houtven et al., 2013). Nevertheless, this pilot study does not distinguish between women who have older, largely independent children living at home and women with young children who need substantial care. It is possible the relationship between stereotype threat and perceived negative career consequences of policy utilization would be more pronounced among women who have infants and toddlers. Study 1 examines this possibility by sampling female employees who recently returned to work after the birth of a child, while also addressing the limitation of relying on a single item measure of the key outcome variable.

## Study 1

Stereotypes about working *mothers* are even more pervasive than those of working women. Working mothers are thought to spend less time at work and consequently are seen as less productive compared to their male counterparts (Heilman and Okimoto, 2008; Wallace and Young, 2008). Mothers are thought to be conserving energy for their family responsibilities, or are perceived as having less energy to expend at work after meeting their domestic responsibilities (Voydanoff, 2004). These stereotypes can make it even more challenging for women to manage their multiple identities (Hodges and Park, 2013), where the expectations of being a good mother conflict with those of being a good employee (Wallace and Young, 2008). Thus, Study 1 sought to provide a conceptual replication of the relationship between stereotype threat and perceived negative career consequences of utilizing family-friendly policies among a sample of mothers who recently returned to work after the birth of a child. Study 1 also used an expanded measure of the perceived negative career consequences of utilizing family-friendly policies to provide a more rigorous test of these ideas.

### Method

#### Participants and Design

Participants were 65 working mothers who recently returned to work after the birth of a child (*M*_age_ = 35.08, *SD*_age_ = 4.82). They were recruited through a newsletter distributed by a local center for mothers and babies in Australia. Women who had recently had a baby were asked to participate in an online survey, at the end of which they could provide an email address to be entered into a prize draw to win a $100 gift card. Participants worked in a range of industries, including healthcare and social assistance (35.4%), professional, scientific, and technical (27.7%), information occupations (7.7%), and retail (6.2%). The majority of respondents (70.7%) worked in organizations that offer family-friendly policies, although 18.5% reported their organizations did not offer family-friendly policies. The remaining participants (9.2%) were not sure whether their organization offered family-friendly policies (1 person did not answer this question). The average tenure with their organization was 6.81 years (*SD* = 5.52), and the average number of children was 1.68 (*SD* = 0.83).

#### Measures

Before completing the survey items, participants were provided with the following explanation: “*Family-friendly policies consist of programs, resources and procedures that organizations have in place to help employees balance work and family responsibilities. Family-friendly policies can include things like flexible working hours and job sharing. We’re interested in your opinions about family-friendly policies more generally, not a particular program.*” In addition to the measures below, participants responded to items unrelated to the central goals of this manuscript. For transparency, the additional constructs measured in Studies 1 and 2 (and their correlations with stereotype threat) are provided in an Appendix. All data are available upon request.

##### Stereotype threat

Stereotype threat was measured on a 7-point scale (1 = *strongly disagree*, 7 = *strongly agree*) using four items from the scale developed by von Hippel et al. (2011a; α = 0.96; e.g., “*Some of my colleagues feel I’m not as committed to my career because I’m a woman*”) and adapted from Steele and Aronson (1995).

##### Perceived negative career consequences of utilizing family-friendly policies

Perceived negative career consequences of utilizing family-friendly policies were assessed on a 7-point scale (1 = *strongly disagree*, 7 = *strongly agree*) using four items (α = 0.93) developed for this study based on previous research. One item (“*Using family-friendly programs would harm my status at work*”) was adapted from Butler et al. (2004). Two items (“*Using family-friendly programs would hurt my career progress*”; “*Using family-friendly programs would suggest that I’m not as serious about my career as employees who don’t use these programs*”) were adapted from Thompson et al. (1999). A final item (“*Using family-friendly programs would result in negative outcomes for me at work*”) was developed for this study.

### Results and Discussion

Consistent with the pilot data, experiences of stereotype threat were associated with more perceived negative career consequences of utilizing family-friendly policies (*r* = 0.28, *p* = 0.029). This effect remained significant when controlling for number of children (β = 0.31, *p* = 0.016).

This study replicated the results in the pilot study showing a positive link between stereotype threat and perceived negative career consequences of utilizing family-friendly policies. This study focused exclusively on working mothers, a sample in which family-friendly policies are likely to be of central importance. Family-friendly policies often target this population, and thus the association between stereotype threat and perceived negative career consequences of utilizing family-friendly policies in this sample is indicative of the potential applied importance of our findings. A limitation of this study is its cross-sectional and correlational design, which does not allow conclusions about causality. Additionally, although women who experience stereotype threat perceive more negative career consequences from utilizing family-friendly policies, these data do not speak to women’s reticence to actually use the policies. Study 2 addressed this unanswered question.

## Study 2

Women who experience stereotype threat are likely to take steps to distance themselves from the stereotype (Steele and Aronson, 1995; von Hippel et al., 2005). This process of distancing oneself from the stereotype may manifest itself in women being less likely to avail themselves of family-friendly work policies. Thus, we hypothesized that to the degree that women experience stereotype threat, they will report less interest in utilizing the very policies that have been designed to help them (Hypothesis 3). In addition, we aimed to replicate the relationship between stereotype threat and perceived consequences of family-friendly policies (Hypothesis 1).

### Method

#### Participants and Design

Participants were 473 working women (*M*_age_ = 30.01, *SD*_age_ = 10.57) in Australia recruited using a convenience sampling approach, including advertising in a university staff newsletter, and forwarding the survey to organizational contacts. In return for their participation, participants were given the chance to win one of several $100 gift cards. Nineteen percent (19.1%) of participants had children living at home, and 60.4% of participants intended to have their first child, or more children, in the future. Of the women who were currently childless, 68.8% reported that they wanted to have children in the future. Participants worked in a number of different industries, most frequently education (25.8%), science and technology (15.8%), mining (7.2%), and health and community services (6.8%). Their average tenure in their current organization was 3.48 years (*SD* = 4.16).

#### Measures

##### Stereotype threat

Stereotype threat (α = 0.87) was assessed using the 4-item measure from Study 1.

##### Perceived negative career consequences of utilizing family-friendly policies

Perceived negative career consequences of utilizing family-friendly policies were assessed using a single item from Thompson et al. (1999) that was used in Study 1: “*Using family-friendly programs would suggest that I’m not as serious about my career as employees who don’t use these programs.*”^5^ Participants responded on a 7-point scale, ranging from 1 = *strongly disagree* to 7 = *strongly agree*.

##### Interest in utilizing family-friendly policies

To measure interest in utilizing family-friendly policies, participants were presented with a list of eight common family-friendly programs and asked how likely they would be to use each program if it were available in their workplace (α = 0.78). Participants responded on a 7-point scale ranging from 1 = *very unlikely* to 7 = *very likely*. The list of common family-friendly programs was adapted from a measure developed by Hammer et al. (2005). The programs included were alternative work arrangements, flexible work hours, job-sharing, telecommuting, unpaid leave, personal time off/paid leave, on-site support groups, and work and family seminars.

### Results and Discussion

Descriptive statistics and correlations are presented in **Table 2**. An independent-groups *t*-test showed no significant difference in stereotype threat between women who did not have children (*M* = 3.00, *SD* = 1.42) and women who had children (*M* = 2.91, *SD* = 1.58), *t*(454) = 0.51, *p* = 0.613, Cohen’s *d* = 0.06. A second independent groups *t*-test showed that there was also no significant difference in stereotype threat between women who were planning to have their first child, or more children, in the future (*M* = 3.07, *SD* = 1.49), and women who were not planning to have children, or to have more children (*M* = 2.87, *SD* = 1.40), *t*(448) = 1.42, *p* = 0.157, Cohen’s *d* = 0.14.

**Table 2:** Descriptive statistics and correlations for Study 2

Variable	1	2	3	4	5	6	*M*	*SD*
1. Stereotype threat	–	0.41^∗∗∗^	0.12^∗^	-0.03	-0.02	0.07	2.99	1.46
2. Perceived negative career consequences of utilizing FFP		–	<0.01	0.07	0.03	-0.02	3.83	1.65
3. Interest in FFP utilization			–	0.06	0.18^∗∗∗^	0.10^∗^	5.15	0.97
4. Age				–	0.42^∗∗∗^	-0.54^∗∗∗^	30.01	10.57
5. Children living at home					–	-0.36^∗∗∗^	-0.62	0.79
6. Intention to have children in future						–	0.21	0.98


*FFP, family-friendly policies. All correlations use pairwise deletion (*N* varies between 384 and 460). Children living at home and intention to have children in the future are coded as -1 = no, 1 = yes. ^∗^*p* < 0.05; ^∗∗^*p* < 0.01; ^∗∗∗^*p* < 0.001.*

In support of Hypothesis 1 and consistent with Study 1, stereotype threat was positively correlated with perceived negative career consequences from using family-friendly policies (see **Table 2**)^6^^,^^7^.

Unexpectedly, there was a positive correlation between stereotype threat and interest in utilizing family-friendly policies, such that women who experienced stereotype threat at work indicated *more* interest in utilizing family-friendly policies (see **Table 2**), despite their beliefs that doing so would hurt their career. Thus, Hypothesis 3 was not supported. The perceived negative career consequences of utilizing family-friendly policies variable was uncorrelated with interest in utilization of family-friendly policies (see **Table 2**).^8^

To explore whether the relationship between stereotype threat and interest in utilization was mediated by perceived consequences, we conducted bootstrapped mediation analyses with 10,000 resamples (using model 4 of the PROCESS macro; Hayes, 2008). There was no significant indirect effect of stereotype threat on interest in utilization through perceived negative career consequences of utilizing family-friendly policies (*IE* = -0.01, *SE* = 0.01, 95% CI: -0.04, 0.01).

This study replicated our previous findings that stereotype threat is positively related to perceived negative career consequences associated with using family-friendly policies. Unexpectedly, however, stereotype threat was positively associated with women’s intentions to utilize work-family practices. Family-friendly policies may be perceived as a double-edged sword among women who experience stereotype threat – women perceive the policies as harmful to their career but feel they have no choice but to use them. Importantly, however, we did not measure actual utilization, nor do we know whether participants had access to family-friendly policies. Nonetheless, these data suggest that women who experience stereotype threat feel the benefits of these policies outweigh the potential career costs associated with using them. In an ideal world, family-friendly policies would be an unmitigated plus, but the unfortunate reality appears to be that among women who experience stereotype threat these policies (are perceived to) incur a cost.

## General Discussion

Employees in organizations that offer family-friendly policies and programs hold more positive job attitudes and are less likely to withdraw from work (Kossek and Ozeki, 1998; Anderson et al., 2002; Kossek et al., 2014). The current research, however, suggests that these benefits may not be realized among women who experience stereotype threat. Across two studies (and a pilot study) with working women, we found that stereotype threat was associated with the perception that taking advantage of family-friendly policies would have negative career consequences. Interestingly, controlling for whether women had children living at home (pilot study and Study 2), or whether they intended to have children in the future (Study 2) did not change any of the results, suggesting that issues surrounding family-friendly policies are relevant to all working women, not just current or future mothers.

### The Disconnect between Perceived Career Consequences and Interest in Policy Utilization

Ironically, Study 2 suggests that, despite perceptions of negative career consequences of utilizing these policies, women who experienced stereotype threat were more interested in using them. Although the current studies do not provide data that help us understand this disconnect, there are several potential explanations. For example, perhaps women experiencing stereotype threat feel the benefits of the policies outweigh the costs associated with using them. If so, family-friendly policies may be perceived as a double-edged sword, whereby women perceive the policies as costly to their career, but feel that the benefits they bring are necessary.

In our previous work, stereotype threat has been associated with work disengagement, including intentions to quit and to retire (e.g., von Hippel et al., 2013). In this sample, the increased interest in using family-friendly policies in the presence of perceived costs may be another signal of disengagement from work. Women who feel stereotype threat may believe they have poor prospects in their career and so disengage from work, taking advantage of family-friendly policies to make life easier in other domains.

### Family-Friendly Policies Can Be Problematic

Our work suggests that, like affirmative action strategies, family-friendly policies may even the playing field in principle, but have negative consequences in practice (Heilman et al., 1992). Previous work examining affirmative action policies found that people perceive the recipients of such policies as in need of help, less committed, and as stereotypic of women in general (Heilman et al., 1992). Our work suggests that working women may be aware of such perceptions, and rightly perceive family-friendly policies as having negative career consequences. Thus, it is particularly important that we better understand what relates to perceptions of negative career consequences of utilizing family-friendly policies, both among people who use them and among those who do not. Additionally, future research should consider how employees perceive their co-workers who make use of family-friendly policies.

### Limitations and Future Directions

As with any research it is necessary to interpret these findings in light of their limitations. Although this research focused on “family-friendly policies,” whether women had children or not did not impact the results. It is possible that all women, irrespective of parent status, must contend with the stereotype that they need to manage work and family. Such a possibility is consistent with research showing that women do more housework than men and are also more likely to provide support for elderly parents (Bianchi et al., 2012; Van Houtven et al., 2013). Future research should attempt to disentangle “family-friendly policies” from other workplace flexible practices.

The field settings of our studies are both a strength and a weakness. On the strength side, participants were working women (many of whom were also mothers) and thus family-friendly policies are consequential in their lives. But working women are busy people, and so in an effort to maximize participation rates we kept the surveys as short as possible. As a consequence, we did not examine various mediators and moderators that might facilitate understanding of the current findings. For example, do women who experience stereotype threat worry that utilizing these policies will signal they are prioritizing family over work? Will the relationship between stereotype threat and perceived career consequences be attenuated by individual (e.g., self-esteem) or work-related resources (e.g., hierarchical position)? The pilot study provides suggestive evidence that perceived organizational support mediates the relationship between gender imbalance at the top of the organizational hierarchy and perceived negative career consequences of policy utilization. This finding is particularly troubling in light of research demonstrating that perceptions of support for such policies can be more important than availability of the policies themselves (Behson, 2005; Wayne et al., 2013). Indeed, organizations find that even when they have desirable family-friendly policies in place, employees often do not have access to these policies (Shrm Survey Findings, 2015). Due to the organization-specific nature of perceived organizational support (e.g., participants working in organizations without family-friendly policies cannot answer a question about perceived support in day-to-day practice) we were unable to pursue this line of inquiry in Studies 1 and 2 (which relied on employees from numerous organization). Thus, further research is required to have confidence in this mediating mechanism.

Although the pilot study manipulated the salience of gender imbalance in the organization to elicit stereotype threat, the remaining studies relied on correlational and cross-sectional designs. Thus, it is possible that stereotype threat is not a causal mechanism in these correlational studies. For example, women who use family-friendly policies may believe utilizing these policies sends the message that they are unable to balance family and work, and thus, as a consequence, might be more susceptible to experiences of stereotype threat. Additionally, an unmeasured variable may account for these relationships. For example, an unwelcoming organizational climate is likely to lead to feelings of stereotype threat as well as beliefs that utilizing family-friendly policies have negative career consequences. This possibility is consistent with Steele’s (1997) theorizing – an unwelcoming climate will lead to feelings of stereotype threat because it suggests to women that they have “poor prospects” to advance their career and causes women to feel a “lack of belonging.” Longitudinal or experimental research designs are required to better understand these relationships.

Traditional gender roles involve women in the role of primary caregiver, and thus women are more susceptible to identity conflict from work than their male counterparts (Hodges and Park, 2013; Williams et al., 2016). For example, the role of “good” mother and wife is very different to that of “good” father and husband (Nomaguchi et al., 2005). The expectations of fatherhood and the demands of work roles often coincide, whereas the expectations of motherhood and work usually conflict (Milkie and Peltola, 1999; Okimoto and Heilman, 2012). For these reasons, our research focused exclusively on women, but future research would benefit from understanding men’s perceptions of these issues. Family-friendly policies are important for men, and research needs to address the potential work-family conflict that men experience. Research suggests men face a “flexibility stigma” whereby utilizing family-friendly policies calls into question their devotion to the job (e.g., Rudman and Mescher, 2013). This stigma might be particularly strong in organizations where there are few women because there is less likely to be a norm of acceptability around using family-friendly policies. More generally, family-friendly policies oriented toward women and not men make it more difficult to change the norm of women as primary caregivers. Finally, future research should examine whether these results generalize to other groups who are susceptible to stereotype threat in the workplace (e.g., older employees; ethnic minority group members).

## Conclusion

Demographic, economic, and egalitarian pressures have coalesced to bring family-friendly policies to the center of many organizations’ staffing practices. Our research suggests that female employees are susceptible to stereotype threat, which in turn is associated with more negative views of family-friendly policies. These results highlight the difficulties faced by companies who offer family-friendly policies, and clarifies the need for organizations to better communicate and promote their policies. Identity safe workplaces are necessary to reduce experiences of stereotype threat, which in turn should reduce perceptions that family-friendly policy utilization has negative career consequences. Although these policies are designed to help employees, there may be perceived costs of utilization in organizational climates where women feel stereotyped. In summary, these findings suggest that the mere provision of family-friendly policies is unlikely to create the kind of family-friendly workplaces that organizations are attempting to provide their employees.

## Ethics Statement

This study was approved by University of Queensland’s School of Psychology Ethics Review Panel (Studies 2 and 3) and University of New South Wales School of Psychology Ethics Review (Study 1). Participants read an information sheet and then chose to continue to the survey or exit the survey (by closing their web browser).

## Author Contributions

CvH and EK designed the studies and coordinated data collection. EK analyzed the data. CvH, EK, and HZ discussed the results and wrote the paper. All authors approved the final submission.

## Conflict of Interest Statement

The authors declare that the research was conducted in the absence of any commercial or financial relationships that could be construed as a potential conflict of interest.

The reviewer LM declared a shared affiliation, though no other collaboration, with the author EK to the handling Editor, who ensured that the process nevertheless met the standards of a fair and objective review.

## Footnotes

1 Assumptions regarding homogeneity of variance were not met [Levene’s *F*(197) = 4.09, *p* = 0.045], and so we report a conservative *t-*statistic (which does not use the pooled statistic for the error term), and adjust the degrees of freedom using the Welch–Satterthwaite method. These results remained non-significant when controlling for parental status (-1 = no children, 1 = children) in an ANCOVA, *F*(1,164) = 2.15, *p* = 0.145, $\eta_{p}^{2}$ = 0.01. Finally, no interaction emerged between the gender imbalance manipulation and parental status in predicting perceived consequences of utilizing family-friendly policies, *F*(15,132) = 0.94, *p* = 0.525, $\eta_{p}^{2}$ = 0.10.

2 As per footnote 2, *t*-values and degrees of freedom were adjusted because assumptions regarding homogeneity of variance were not met. These effect remained significant when controlling for parental status (-1 = no children, 1 = children) in an ANCOVA, *F*(1,164) = 8.97, *p* = 0.003, $\eta_{p}^{2}$ = 0.05.

3 We also ran this model including parental status and the parental status × gender imbalance manipulation as covariates, and found the same indirect effect of the gender imbalance reminder through stereotype threat (*IE* = 0.09, *SE* = 0.04, 95% CI: 0.02, 0.20).

4 This indirect effect remained when controlling for parental status and the parental status × gender imbalance manipulation interaction (*IE* = -11, *SE* = 0.06, 95% CI: -0.25, -0.03).

5 A second item from Thompson et al. (1999) was included in the survey and can be seen in the Appendix. This item is not about family-friendly policies and thus is not included in these analyses. Nonetheless, the results remain unchanged when both items are included in the analyses.

6 As noted in the Appendix, this study also measured gender identification, and thus we are able to explore whether, consistent with previous laboratory research, gender identification moderates these relationships. The interaction between gender identification and stereotype threat does not predict the perceived consequences of family-friendly policies (β = 0.03, *p* = 0.556) or interest in utilizing family-friendly policies (β = 0.05, *p* = 275). McClelland and Judd (1993) note that power to detect an interaction in a field study is much lower than in traditional lab studies, and thus this null effect should be interpreted with caution.

7 To control for parental status we conducted a hierarchical multiple regression analysis in which we entered parental status (-1 = no children, 1 = children) at the first step, and stereotype threat at the second step, to predict perceived negative career consequences. This analysis showed that the stereotype threat – career consequences relationship was also present when controlling for parental status (β = 0.42, *p* < 0.001). We conducted a second hierarchical multiple regression analysis in which we entered intention to have (more) children (-1 = no intention to have (more) children, 1 = intent to have (more) children in the future) at step one, and stereotype threat at the second step, to predict negative career consequences. The stereotype threat – career consequences relationship remained present when controlling for intention to have (more) children in the future (β = 0.41, *p* < 0.001). Finally, we also conducted two hierarchical multiple regression analyses in which stereotype threat and parental status, or stereotype threat and intention to have (more) children were entered at the first step, and their interaction at the second step. Consistent with the pilot study, there was no significant interaction between stereotype threat and parental status (β = 0.00, *p* = 0.998), or between stereotype threat and intentions to have children (β = 0.04, *p* = 0.556), in predicting perceived career consequences.

8 To control for parental status we conducted a hierarchical multiple regression analysis in which we entered parental status at the first step, and stereotype threat at the second step, to predict perceived interest in utilization. We found that the stereotype threat – utilization relationship was also present when controlling for parental status (β = 0.13, *p* = 0.005). We conducted a second such analysis including intention to have (more) children in the future, instead of parental status. We also found that the stereotype threat-utilization relationship remained significant in this analysis (β = 0.12, *p* = 0.011). Finally, and consistent with the prior results, there was no interaction between stereotype threat and parental status (β = 0.03, *p* = 0.533), or stereotype threat and intention to have children (β = 0.05, *p* = 0.556), in interest in utilizing family-friendly policies.
