# Supplementary material for: Stereotype Threat and Perceptions of Family-Friendly Policies among Female Employees
Source: Front Psychol. 2017 Jan 5;7:2043. doi: 10.3389/fpsyg.2016.02043 (PMC5216670; doi:10.3389/fpsyg.2016.02043)
Supplement: Supplementary file 1 [file Data_Sheet_1.docx]

**Appendix: Data Transparency Table**

| **Variable** | **Correlation with Stereotype threat** |
| --- | --- |
| Study 1: work-family conflict | *r* = .30, *p* = .018 |
| Study 1: family-work conflict | *r* = .36, *p* = .004 |
| Study 2: gender identification | *r* = .08, *p* = .087 |
| Study 2: family-friendly policy fairness | *r* = .04, *p* = .392 |
| Study 2: Turning down a promotion or transfer for family-related reasons will seriously hurt one’s career progress. | *r* = .32, *p* < .000 |
